# Fitnesser’s Intrinsic Motivations of Green Eating: An Integration of Theory of Planned Behavior and Hedonic-Motivation System Adoption Model

**DOI:** 10.3389/fpsyg.2021.670243

**Published:** 2021-05-21

**Authors:** Yuan Chen, Bey-Fen Lee, Yen-Cheng Lu

**Affiliations:** ^1^Hubei University of Technology Engineering and Technology College, Wuhan, China; ^2^School of Management, Wuhan University of Technology, Wuhan, China; ^3^Department of Hospitality Management, Chung Hwa University of Medical Technology, Tainan, Taiwan; ^4^Department of Sport, Health, and Leisure, Chung Hwa University of Medical Technology, Tainan, Taiwan

**Keywords:** intrinsic motivation, green eating, theory of planned theory, hedonic-motivation system adoption model, social recognition, environmental ethics

## Abstract

Global climate change arouses people’s attention to environmental protection and, therefore, changes consumption habits. Food overconsumption not only produces extra waste but also pollutes the environment. Therefore, it is important to understand the factors that motivate people to eat green, an eco-friendly way to consume food. To keep the body in good shape, the fitnessers concern more about diet than the general people. This study explored intrinsic motivations, such as social recognition, environmental ethics, curiosity, joy of purchase, perceived usefulness, subjective norm, and perceived behavior control as constructs that affect fitnesser’s green eating intention. All constructs except curiosity have significant impacts on behavior intention. The results demonstrate that social recognition and environmental ethics have significant effects on curiosity, joy of purchase, perceived usefulness, subjective norm, and perceived behavior control. The mediation effects between social recognition and behavior intention are not supported. The mediators between environmental ethics and behavior intention are joy of purchase, perceived usefulness, subjective norm, and perceived behavior control.

## Introduction

With the rapid growth of the world’s population and the increasing consumption of energy, environmental pollution caused by energy consumption has attracted wide attention. China has a population of 1.3 billion, accounting for 18.57% of the world’s population. Also, China is the world’s second largest economy (Barboza, 2010), and its food consumption and role in environmental protection cannot be ignored. Food is a basic need for human life and cannot be substituted. Food consumption accounts for 20–30% of the environmental impact in the West (Tukker and Jansen, 2006).

Fast economic development not only brings people a better life but also causes the environment a heavy burden. To prevent the environment from being destroyed by increasing pollution, the government supported various green industries and encouraged people’s green behaviors (Steg and Vlek, 2009). One of the behaviors is green eating, which is consuming food in an eco-friendly way. Key elements about eating green include eating locally grown foods, choosing organic foods if possible, limiting intake of processed or fast foods, and consuming meatless meals weekly (Weller et al., 2014). Green eating behavior can reduce energy consumption by not buying food in a distance and lessen waste production by eating fresh or organic food instead of processed food. For sustainable development, it is important to implement environmental protection education to the society, so as to understand the factors that influence green eating intention (Muposhi and Dhurup, 2017; Kim and Kim, 2018; Dalvi-Esfahani et al., 2020; Lacroix and Gifford, 2020).

Fitness has a broader meaning in Eastern countries. Not limited to bodybuilding, a fitness center could provide aerobics, sports, group classes, pools, steam rooms, Jacuzzis, saunas, or even massage rooms or lounge areas for social interaction. To maintain their body figure, fitnessers believe exercise can prevent aging, and selected diet can increase physical strength or even enhance immunity. For example, they tend to cut down on high sugar or starchy foods and eat high-fiber or low-calorie foods. Therefore, their awareness of food selection is more cautious than the general public. This study adopted the theory of planned behavior (TPB) to find out the determinants including social recognition, environmental ethics, curiosity, joy of purchase, perceived usefulness, subjective norm, and perceived behavior control to understand the impacts on fitnesser’s intention of green eating.

Theory of planned behavior is an extensively applied research model in the intention for green eating (e.g., Mundorf et al., 2018; Ahmad et al., 2020; Canova et al., 2020; Carfora et al., 2020; Dalvi-Esfahani et al., 2020; Malan et al., 2020; Ruangkanjanases et al., 2020). However, prior studies have adopted or extended TPB in several aspects for predicting intention toward green eating, and there are still two major limitations that remain to be solved. First, few of the studies have elaborated on how antecedents affect the key components for green eating. These limitations drive the possibilities for further exploration in green behaviors. Therefore, this research applied TPB as a basic framework and further attempted to include a cognitive construct (i.e., social recognition, environmental ethics, curiosity, joy of purchase, and perceived usefulness) to measure its effect on intention toward green eating. Second, because intrinsic motivation has a greater impact on human behavior than extrinsic motivation (Csikszentmihalyi, 1990; Thomas and Velthouse, 1990), unlike the other scholars who apply only TPB in their studies, this study explored TPB, the hedonic-motivation system adoption model (HMSAM), and intrinsic constructs including social recognition and environmental ethics in the proposed model to examine fitnesser’s acceptance of green eating.

## Literature Review and Theoretical Background

### Theory of Planned Behavior

According to the theory of reasoned action (TRA), one’s behavior is based on his behavior intention, and one’s behavior intention is affected by his attitude and subjective norm (Fishbein and Ajzen, 1975). Originating from the TRA, TPB extends the TRA with perceived behavioral control as an antecedent variable of behavioral intention (Ajzen, 1991). TPB has been extensively adopted in explaining human behavior intention, not to mention environmental protection issues.

A number of studies agreed that TPB predicted different behaviors *via* external variables and antecedents (e.g., Chen and Hung, 2016; Paul et al., 2016; Hsu et al., 2017; Al-Jubari, 2019; Sun et al., 2019; Hoque and Hossan, 2020; Parash et al., 2020; Si et al., 2020; Uzun and Kilis, 2020). A meta-analysis conducted by Hagger et al. (2002) confirmed that the three aforesaid factors can be used to predict behavioral intentions and behavior. Therefore, this study hypothesized H_1_ and H_2_ as follows:

**Hypothesis 1 (H_1_).** *Fitnesser’s subjective norm is positively correlated with their intention to eat green.*

**Hypothesis 2 (H_2_).** *Fitnesser’s perceived behavior control is positively correlated with their intention to eat green.*

### Hedonic-Motivation System Adoption Model

van der Heijden (2004) proposed his research result regarding hedonic-motivation system (HMS) by adding joy in the technology acceptance model (TAM) as a mediator between perceived ease of use and behavioral intention to use. Prior research indicated the influence of perceived usefulness on behavioral intention (e.g., Chen et al., 2009, 2013; Tsai et al., 2020). Moreover, empirical evidence also supported the relationship between perceived usefulness and purchase intention (e.g., Jamal and Sharifuddin, 2015; Moslehpour et al., 2018).

Grounded in flow-based cognitive absorption (CA), Lowry et al. (2012) improved the HMS by integrating intrinsic motivations, such as curiosity and joy in the HMSAM. Oluwajana et al. (2019) found that curiosity, joy, and perceived usefulness have a direct impact on behavior intention based on HMSAM. Past studies also provided empirical evidence linkages between behavioral intention intrinsic motivations (i.e., curiosity, joy, and perceived usefulness) (e.g., Koo and Choi, 2010; Shu, 2014; Mohammadi, 2015). According to the above discussion, this study proposed H_3_, H_4_, and H_5_ as follows:

**Hypothesis 3 (H_3_).** Fitnesser’s curiosity is positively correlated with their intention to eat green.

**Hypothesis 4 (H_4_).** Fitnesser’s joy of purchase is positively correlated with their intention to eat green.

**Hypothesis 5 (H_5_).** Fitnesser’s perceived usefulness is positively correlated with their intention to eat green.

### Social Recognition

Social recognition is about public acknowledgment of people’s status, merits, or personality (Susewind and Walkowitz, 2020). Maslow’s (1970) hierarchy of needs classifies human needs into five levels to motivate human behavior. Social recognition serves as a postconsumption feedback on the viability of this social function (Fischer et al., 2010; McPhail et al., 2011; Stead et al., 2011). Thus, fitnessers use environmentally friendly products, and the sense of society can satisfy their need for status, achievement, recognition, and self-esteem. Similarly, in prior empirical evidence, social recognition is one of the influential antecedents in the formation of individual perceptions (e.g., Chuang and Dellmann-Jenkins, 2010; Kim et al., 2016). Therefore, this study proposed the following hypotheses:

**Hypothesis 6 (H_6_).** Fitnesser’s social recognition is positively correlated with the subjective norm to eat green.

**Hypothesis 7 (H_7_).** Fitnesser’s social recognition is positively correlated with the perceived behavioral control to eat green.

Martella et al. (2015) identified various human needs including social recognition and curiosity based on the gamification framework. To develop a successful constructivist-based learning environment, several issues including curiosity and social recognition that motivate students were the top priority (Herring, 2004).

As mentioned in the social attraction theory, society members who reflect the collective group standard may appear more attractive by other group members (Hogg and Hardie, 1991) and, hence, be beneficial from higher levels of social recognition, in which their subjective well-being is positively affected. Likewise, in a cross-country study regarding religious people, social recognition has a positive impact on happiness (Stavrova et al., 2013). Besides, prior research also found the linkage between social recognition and perceived usefulness (Wu and Chen, 2017). Therefore, our study proposed the following hypotheses:

**Hypothesis 8 (H_8_).** Fitnesser’s social recognition is positively correlated with the curiosity to eat green.

**Hypothesis 9 (H_9_).** Fitnesser’s social recognition is positively correlated with the joy to eat green.

**Hypothesis 10 (H_10_).** Fitnesser’s social recognition is positively correlated with the perceived usefulness to eat green.

### Environmental Ethics

Environmental ethics ensures a healthy man–nature relationship (Misra, 1995). In the beginning, the importance between humans and nature has been ignored, but now, humans have a new understanding of ecosystems and of keeping the balance of the cultural and biological diversity of humans and other forms of life (Leopold, 1949; Ellis and Ramankutty, 2008; Leopold et al., 2010). Including the extent of human decent obligations to the environment, environmental ethics focuses on the collective action of human beings on nature (Holden, 2005). Humans and other creatures form an interdependent system on Earth, and humans are not superior to other creatures (Taylor, 1986). Rolston (2020) refers to the basis of environmental ethics as natural love of an individual, and the original experience might be hedonic.

In a recent study in the United Kingdom regarding small and medium enterprise (SME) owners, North and Nurse (2014) discovered that they have strong moral and environmental ethics in running “normal” businesses while having curiosity about the problem of climate change and its solutions. Therefore, the concerns of environmental issues have been prevailed to the “normal” citizen. McShane (2007) stated that environmental ethics should not give up on intrinsic value, like joy. On the enjoyment of life, Chinese fitnessers have become more and more concerned about the impact of ecological degradation, and advertisement emphasizing eco-friendliness appears to having significant impact on their perception of perceived usefulness and credibility (Chan, 2004). Therefore, this study proposed the following hypotheses:

**Hypothesis 11 (H_11_).** Fitnesser’s environmental ethics is positively correlated with the curiosity to eat green.

**Hypothesis 12 (H_12_).** Fitnesser’s environmental ethics is positively correlated with the joy to eat green.

**Hypothesis 13 (H_13_).** Fitnesser’s environmental ethics is positively correlated with the perceived usefulness to eat green.

In the study of food consumption (Kim, 2014), ecological concern refers to the concern about doing the right thing for animal welfare, the environment, and the ecosystem, which can be regarded as moral norms. Human should respect the environment *via* environmental ethics and beliefs and ensure the ethical connection between humans and the environment. These should also dominate the mind, as well as attitudes and behaviors (Chen and Hung, 2016). In this context, similar to the viewpoints of Leopold (1949), Taylor (1986), and Arvola et al. (2008), environmental ethics is significantly related to the three aforementioned factors in the TPB. Studies on ethical consumerism like Shaw and Shui (2002) used the TPB to explain customers’ behavioral intentions to purchase ethically produced goods. Therefore, the study proposed the following hypotheses:

**Hypothesis 14 (H_14_).** Fitnesser’s environmental ethics is positively correlated with the subjective norm to eat green.

**Hypothesis 15 (H_1__5_).** Fitnesser’s environmental ethics is positively correlated with the perceived behavior control to eat green.

**FIGURE 1:**
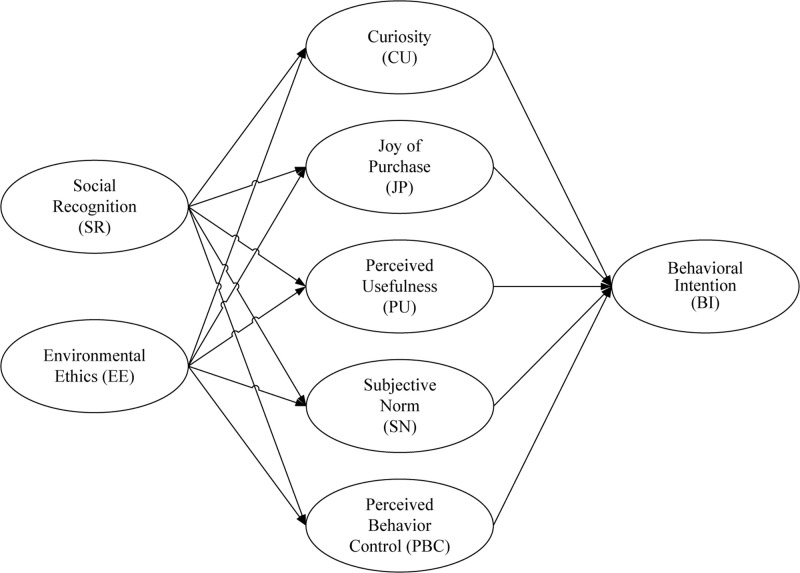
The research model.

According to the literature review and the hypotheses development, this study proposed a research model as shown in Figure 1.

## Research Method

Measurement scales for all constructs in this study were designed based on previous studies and were scored on a seven-point Likert scale, with higher scores indicating higher agreement with the question. Behavioral intention, subjective norm, and perceived behavioral control were adapted from Ajzen (1991), Paul et al. (2016), and Yadav and Pathak (2016). Perceived usefulness with three items was adopted from Chen et al. (2013) and Wu et al. (2015). Curiosity and joy with three items, respectively, were modified from Agarwal and Karahanna (2000) and Lowry et al. (2012). Social recognition with three scales was developed from Fischer et al. (2010) and McPhail et al. (2011). Finally, environmental ethics had three items adopted from Henriques and Sadorsky (1999). Before the formal questionnaire was sent out, a pilot test was conducted in order to revise the content of the questionnaire to avoid any discrepancies. Therefore, the content validity of this research could be improved, eliminating the occurrence of ambiguous words and inappropriate questioning through the pilot test.

The questionnaire was made available at a professional and famous online survey website^1^ and announced to the public in four major cities—Beijing, Shanghai, Guangzhou, and Shenzhen in China. The beginning of the questionnaire briefly explains the definition of green eating, and the first question determines whether the respondent has the experience of green eating. Only fitnessers with green eating experience were invited to participate in the survey and were instructed to answer all questionnaire items.

Beginning in early July 2020, the data collection process lasted for 3 weeks. To improve external validity, we collected data only from active fitnessers, rather than from the general public. After removing seven invalid questionnaires, 786 valid responses were analyzed by AMOS 24. Among the samples of this study, 47.80% were male and 52.20% were female. Furthermore, 16.93% were aged under 25 years, 61.31% were 26–45 years, 18.35% were 46–65 years, and 3.41% were above the age of 66 years.

## Analysis Results

### Measurement Model

#### Construct Validity

This study followed the two-step approach of structural equation modeling (SEM) proposed by Anderson and Gerbing (1988) to estimate the measurement and structural model. We chose SEM as the statistical method and AMOS as the analysis tool for two reasons. First, SEM is a family of statistical procedures that could handle the confirmatory factor analysis (CFA) and path analysis. Particularly, CFA is an important procedure to measure construct validity and the path analysis is adopted to evaluate the research hypotheses (Byrne, 2001; Kline, 2011). Second, AMOS is one of the recommended analysis tools to perform the results of SEM (Babin et al., 2008). The first step examined construct reliability and validity of the measurement model using CFA, and the second step checked the path effects and their significance of the structural model. The measurement model was assessed by using the maximum likelihood estimation (MLE) in terms of factor loadings, reliability of measurement, convergent validity, and discriminant validity.

As shown in Table 1, all standardized factor loadings of questions are from 0.699 to 0.894. All composite reliability of the constructs ranging from 0.839 to 0.913 and Cronbach’s alpha ranging from 0.828 to 0.913 exceed 0.7 as recommended by Nunnally and Bernstein (1994), indicating that all constructs have internal consistency. Lastly, the average variance extracted (AVE) of the constructs ranging from 0.633 to 0.777 exceed 0.5 as suggested by Fornell and Larcker (1981) and Hair et al. (2010), showing that all constructs have adequate convergent validity. These results demonstrated that all measurement items had convergent validity.

**TABLE 1:** Results of the measurement model.

**Construct**	**Item**	**Standardized factor loading**	**Construct reliability**		**Convergent validity**
			**Alpha**	**CR**	**AVE**
SR	SR1	0.867	0.902	0.921	0.756
	SR2	0.894			
	SR3	0.847			
EE	EE1	0.803	0.828	0.837	0.633
	EE2	0.699			
	EE3	0.875			
CU	CU1	0.805	0.850	0.850	0.655
	CU2	0.813			
	CU3	0.809			
JP	JP1	0.833	0.872	0.872	0.695
	JP2	0.828			
	JP3	0.839			
PU	PU1	0.816	0.843	0.844	0.644
	PU2	0.764			
	PU3	0.826			
SN	SN1	0.847	0.876	0.877	0.704
	SN2	0.861			
	SN3	0.809			
PBC	PBC1	0.808	0.838	0.839	0.635
	PBC2	0.826			
	PBC3	0.755			
BI	BI1	0.886	0.913	0.913	0.777
	BI2	0.887			
	BI3	0.872			

*Alpha, Cronbach’s alpha; CR, composite reliability; AVE, average variance extracted; SR, social recognition; EE, environmental ethics; CU, curiosity; JP, joy of purchase; PU, perceived usefulness; SN, subjective norm; PBC, perceived behavioral control; BI, behavioral intention.*

As shown in Table 2, the bold numbers in the diagonal direction represent the square roots of AVEs and the off-diagonal numbers are correlations among constructs. Most of the numbers in the diagonal direction are greater than the off-diagonal numbers, and all the correlations are lower than 0.85; therefore, discriminant validity appears to be adequate for this study.

**TABLE 2:** Discriminant validity of the measurement model.

	**SR**	**EE**	**CU**	**JP**	**PU**	**SN**	**PBC**	**BI**
SR	**0.869**							
EE	0.589	**0.796**						
CU	0.569	0.648	**0.809**					
JP	0.660	0.748	0.704	**0.834**				
PU	0.562	0.691	0.719	0.797	**0.802**			
SN	0.634	0.640	0.559	0.746	0.662	**0.839**		
PBC	0.616	0.704	0.633	0.694	0.668	0.702	**0.797**	
BI	0.566	0.748	0.551	0.683	0.622	0.604	0.622	**0.881**

*The items on the diagonal in bold represent the square roots of the AVE; off-diagonal elements are the correlation estimates. SR, social recognition; EE, environmental ethics; CU, curiosity; JP, joy of purchase; PU, perceived usefulness; SN, subjective norm; PBC, perceived behavioral control; BI, behavioral intention.*

#### Detection of Common Method Bias

Preventive measures in this research were taken in order to reduce the common method bias (CMB) that may result from sample collection of a single respondent’s cognitive information by self-reported measurement items and decrease the impact of CMB. In addition to anonymous surveys, this research attempted to hide the meaning of each question and separate measurement items for different constructs as much as possible. The variable results in Tables 1, 2 had an appropriate degree of construct validity, which also indicated that the results were not largely affected by CMB. In addition, this study adopted Harman’s single-factor test to evaluate the severity of CMB (Podsakoff et al., 2003). The model fit of the CFA for the 24 measurement items in this study was better than the single-factor model of CFA significantly. This result could be seen that the impact of CMB was not serious in this research.

### Structural Model Analysis

By using the maximum likelihood method, this study performed structural model testing to estimate the hypothesized relationships of the proposed model. Model fit indicators determine the degree of whether the sample data fit the structural equation model. Schumacker and Lomax (2010) and Kline (2011) recommended a variety of criteria to determine the model fit of a structural model. Jackson et al. (2009) suggested that the commonly used model fit reporting guidelines are *χ*^2^, *df*, *χ*^2^/*df* ratio, GFI, RMSEA, SRMR, CFI, and TLI.

Table 3 demonstrates several model fit indicators and the thresholds recommended by previous studies. Except for *χ*^2^, all model fit indicators exceed the recommended levels suggested by Schumacker and Lomax (2010). Because *χ*^2^ is very sensitive to a large sample, the ratio of *χ*^2^ to its degree of freedom was computed. For a good model fit, the ideal ratio should be below three. Instead of evaluating each index independently, Hu and Bentler (1999) proposed that more strict combination rules should be applied to model fit indices so the type I errors could be controlled. The model fit indicators satisfy most of the independent level of recommended fits and the combination rule. Thus, it has been proven that the proposed model of most of the constructs has a good fit.

**TABLE 3:** Model fit.

**Model fit**	**Criteria**	**Measurement model**	**Structural model**
Chi-squared/*df*	<5	2.848	3.914
GFI	>0.9	0.934	0.903
CFI	>0.9	0.969	0.948
TLI	>0.9	0.962	0.940
NFI	>0.9	0.953	0.932
IFI	>0.9	0.969	0.949
RMSEA	<0.08	0.049	0.061
SRMR	<0.08	0.029	0.039

*Chi-squared/*df*, chi-squared divided by degrees of freedom; GFI, goodness-of-fit index; CFI, comparative fit index; TLI, Tucker–Lewis index; NFI, normed fit index; IFI, incremental fit index; RMSEA, root-mean-square error of approximation; SRMR, standardized root-mean-square residual.*

The results support the research hypotheses regarding the validity of the research model (as shown in Table 4); 54.3% of CU can be explained by SR and EE constructs, 74.2% of JP can be explained by SR and EE constructs, 64.7% of PU can be explained by SR and EE constructs, 62.8% of SN can be explained by SR and EE constructs, 62% of PBC can be explained by SR and EE constructs, and 54% of BI can be explained by CU, JP, PU, SN, and PBC constructs.

**TABLE 4:** Structural model analysis.

**DV**	**IV**	**Unstd. regression weight**	**SE**	***t*-Value**	**Std. path coefficient**	***R*^2^**
CU	SR	0.145	0.042	3.447	0.163***	0.580
	EE	0.689	0.057	12.166	0.648***	
JP	SR	0.191	0.040	4.746	0.202***	0.783
	EE	0.840	0.056	14.893	0.744***	
PU	SR	0.082	0.045	1.848	0.065	0.685
	EE	0.859	0.062	13.844	0.769***	
SN	SR	0.261	0.043	6.030	0.275***	0.613
	EE	0.659	0.056	11.795	0.580***	
PBC	SR	0.183	0.041	4.454	0.206***	0.650
	EE	0.699	0.054	12.896	0.660***	
BI	CU	0.036	0.048	0.741	0.036*	0.549
	JP	0.332	0.063	5.252	0.357***	
	PU	0.126	0.055	2.278	0.134*	
	SN	0.105	0.046	2.274	0.114*	
	PBC	0.200	0.054	3.712	0.201***	

DV, dependent variable; IV, independent variable; SR, social recognition; EE, environmental ethics; CU, curiosity; JP, joy of purchase; PU, perceived usefulness; SN, subjective norm; PBC, perceived behavioral control; BI, behavioral intention. *p-value < 0.05. ***p-value < 0.001.

### Mediation Effects

Bootstrapping mediation analysis can provide confidence intervals to examine the indirect effects. One of the preferred bootstrapping mediation analysis methods is bias-corrected bootstrapping (Williams and MacKinnon, 2008), which is used in this study. As shown in Table 5, the total effect SR→BI, *p* > 0.05, bias-corrected confidence interval (CI) does include zero [CI of SR→BI = (–0.051, 0.448)]. The existence of total effect was not supported. It was not necessary to test the mediation effect. The total effect ER→BI, *p* < 0.05, bias-corrected CI does not include zero [CI of ER→BI = (0.281, 0.919)]. The existence of total effect was supported. The indirect effect EE→CU→BI, *p* > 0.05, both bias-corrected CI includes zero [CI of EE→CU→BI = (–0.036, 0.163)]. Consequently, the hypothesis of the existence of indirect effect was not supported. The indirect effect EE→JP→BI bias-corrected CI does not include zero [CI of EE→JP→BI = (0.094, 0.652)]. Thus, the hypothesis of the existence of indirect effect was supported. The indirect effect ENV→PU→BI bias-corrected CI does not include zero [CI of EE→PU→BI = (0.007, 0.405)]. Therefore, the hypothesis of the existence of indirect effect was supported. The indirect effect ENV→SN→BI bias-corrected CI does not include zero [CI of ENV→SN→CU = (0.007, 0.25)]. Consequently, the hypothesis of the existence of indirect effect was supported. The indirect effect ENV→PBC→BI bias-corrected CI does not include zero [CI of ENV→PBC→BI = (0.037, 0.401)]. Accordingly, the hypothesis of the existence of indirect effect was supported.

**TABLE 5:** Analysis of mediation effects.

**Parameter**	**Estimate**	**Lower**	**Upper**	***p-*Value**
SR→CU→BI	0.005	–0.008	0.046	0.373
SR→JP→BI	0.063*	0.009	0.177	0.015
SR→PU→BI	0.010	–0.006	0.076	0.216
SR→SN→BI	0.028*	0.000	0.088	0.043
SR→PBC→BI	0.036*	0.005	0.111	0.014
EE→CU→BI	0.025	–0.049	0.148	0.464
EE→JP→BI	0.279**	0.114	0.633	0.001
EE→PU→BI	0.010	–0.006	0.076	0.216
EE→SN→BI	0.028*	0.000	0.088	0.043
EE→PBC→BI	0.016	–0.015	0.086	0.224

SR, social recognition; EE, environmental ethics; CU, curiosity; JP, joy of purchase; PU, perceived usefulness; SN, subjective norm; PBC, perceived behavioral control; BI, behavioral intention. *p-value < 0.05. **p-value < 0.01.

## Conclusion

### Theoretical Contributions

Due to the complex nature of sustainability, it is common for researchers to focus only on the economic aspect of environmental solutions and implications. However, this study explored fitnesser’s intrinsic motivations of green eating. Most of the environmental studies suggested to address the extension of TPB in interpreting human behavior in ecological protection issues (e.g., Bagheri et al., 2019; Suki and Suki, 2019; Si et al., 2020). Therefore, integrating only one theory is not sufficient to explain complicated human behavior. This study proposed a research model incorporating TPB and HMSAM to discuss the compact of social recognition and environmental ethics on fitnesser’s green eating intention. In addition, the mediation effects of curiosity, joy, perceived usefulness, subjective norm, and perceived behavior control were investigated. The results supported hypotheses H_1_ to H_10_. Both fitnessers’ social recognition and their environmental ethics are positively correlated with the HMSAM constructs such as curiosity, joy of purchase, and perceived usefulness and the TPB constructs like subjective norm and perceived behavior control.

Social recognition refers to the positive response of society to individual social behavior. The praise and recognition of others is helpful to promote one’s social status. In this study, fitnessers with a higher level of social recognition tend to have a higher curiosity in understanding how green eating can protect environment, have greater pleasure when eating green, and have a better understanding of the usefulness of green eating in ecological protection.

Environmental ethics is the study of environmental issues from an ethical perspective. Since environmental protection has become a manifestation in today’s society, many fitnessers are committed to environmental protection initiatives and are proud of being environmentalists. They are eager to learn more about environmental protection and hope to protect the environment better. They “feel better right now” when they have hedonic goals (Lindenberg and Steg, 2007). They feel that they are doing their part for environmental protection when they eat green and, therefore, feel happy inside.

Fitnessers with a rich knowledge of environmental protection may gain social recognition from their friends. On the other hand, to obtain social recognition, they want to know more or be curious about the mechanism between green eating and environmental protection. When they are involved in these issues, they have full understanding about what green eating can do to the environment. Consequently, they feel that eating green is useful for ecological protection and they are happy to do it in their daily lives. This explained the correlation between social recognition and joy of purchase. Similarly, fitnessers with higher environmental ethics have no doubt in their mind that green eating is useful for environmental protection.

Subjective norm is the perception of the social pressures that individuals experience when they take a particular behavior. It is not surprising that a consumer with a higher social recognition has a higher subjective norm. Fitnessers are possible to cater to their friends to gain higher social recognition, so they will not perform a certain behavior. In other words, they will comply with their friends, to follow the subjective norm, because it is important to raise their social recognition.

As mentioned above, people with higher social recognition have the tendency to cope with their friends better. That is, they have better control of themselves whether or not to perform a particular behavior. Similarly, if fitnessers have higher environmental ethics, they expect to be able to master their behavior and will not do anything that is harmful to the environment. This explained why fitnessers with a higher level of environmental ethics tend to have a higher joy of purchase. Therefore, both social recognition and environmental ethics have a positive impact on perceived behavior control.

This study not only explores the influence of social recognition and environmental ethics on behavior intention, but also discusses the mediation effects of curiosity, joy of purchase, perceived usefulness, subjective norm, and perceived behavior control. Based on the mediation effect analysis, since the total effect of social recognition to behavior intention is not significant, constructs between social recognition and behavior intention are not discussed. However, the total effect of environmental ethics to behavior intention is significant, and the mediation effects of joy of purchase, perceived usefulness, subjective norm, and perceived behavior are supported. In other words, except for the H_11_ that fitnesser’s curiosity is positively correlated with their intention to eat green being not supported, H_12_–H_15_ dealing with environmental ethics having impacts on the behavior intention through the four abovementioned mediators are all sustained.

Intrinsic value is the degree to which an activity is considered to be personally enjoyable (Chiu and Wang, 2008; Jiang et al., 2020). Straume and Vittersø (2012) claimed that the individuals’ feelings of joy or pleasure could affect their behavior. A number of empirical studies have also confirmed the positive effect of hedonic values on consumer behavior intention. Hedonic perception influenced fitnesser’s intentions of adoption significantly (Song, 2014). Choi and Kim (2016) found that both enjoyment and perceived usefulness affected behavioral intention positively. Similarly, in this study, perceived usefulness mediated behavior intention. This result is supported by Chang et al. (2005) in their search of quality antecedents. In a web learning tools study, Lai (2017) found that perceived behavior control mediated behavior intention, and so did the subjective norm. In a study of genetically modified foods, Kim et al. (2014) pointed out that subjective norm and perceived behavior control are positive determinants of behavior intention, though this research proves that both subjective norm and perceived behavior control are mediators between environmental ethics and behavior intention.

### Managerial Implications

There are four theoretical contributions of this article. First, this research combines TPB and HMSAM to extend the research on green eating and to build up the research model from social recognition, environmental ethics, and the two aforesaid models. Second, there is no prior research using the HMS adoption model in discussing fitnesser’s intention in green eating. This study proves that joy of purchase and perceived usefulness positively affect fitnesser’s behavior intention that has filled up the research gap. Third, this study indicates that the relationship between environmental ethics and behavior intention is mediated by joy of purchase, perceived usefulness, subjective norm, and perceived behavior control. Fourth, arousing fitnesser’s social recognition and environmental ethics is helpful in increasing their intention of green eating. Unlike many previous studies that only focused on interpreting consumer behavior with TPB, this research raises the research domain to a different level by integrating social recognition and environmental ethics into the research model.

There are three practical contributions of this study. First, this study validates that increasing fitnesser’s social recognition and environmental ethics can not only raise their joy of purchase, perceived usefulness, subjective norm, and perceived behavior control but also improve their behavioral intention toward green eating. If manufacturers want to increase their sales on green eating, other than increase fitnesser’s social recognition and environmental ethics, they have to enhance fitnesser’s abovementioned four elements to change fitnesser’s behavior intention to eat green.

Second, fitnesser’s joy of purchase, perceived usefulness, subjective norm, and perceived behavior control should be promoted so their intention to eat green will be influenced. Because the mediation effects of the said four constructs are significant in this study, companies can change the fitnesser’s behavior intention to eat green if their aforesaid variables can be improved.

Third, this study demonstrates that fitnesser’s social recognition and environmental ethics are positively associated with joy of purchase, perceived usefulness, subjective norm, and perceived behavior control and are also positively associated with fitnesser’s behavior intention. Both fitnesser’s social recognition and environmental ethics indirectly affect fitnesser’s behavior intention to eat green positively *via* joy of purchase, perceived usefulness, subjective norm, and perceived behavior control.

### Research Limitations and Future Work

Although this study provided some useful insights and viewpoints, it had several limitations and should be addressed in further research. First, the aim of this study was to determine the constructs that influence fitnesser’s behavior intention of green eating. Future research could try to integrate different theories and constructs to better interpret fitnesser’s behavior intention in comparison with this study. Second, the sample collection and research design of this study were undertaken in China. Future research could broaden the sample collection by adding samples from other countries or areas. Multiculture comparison of consumer behavior intention could be explored if possible. Finally, this study deployed a questionnaire survey that only provided cross-sectional data. The concept of environmental protection and the innovation of green eating may change over time; therefore, future research should try to conduct a longitudinal study to reveal the different effects of social recognition and environmental ethics on fitnesser’s behavior intention in different time periods.

## Data Availability Statement

The original contributions presented in the study are included in the article/supplementary material, further inquiries can be directed to the corresponding author/s.

## Ethics Statement

Ethical review and approval was not required for the study on human participants in accordance with the local legislation and institutional requirements. Written informed consent for participation was not required for this study in accordance with the national legislation and the institutional requirements.

## Author Contributions

All authors listed have made a substantial, direct and intellectual contribution to the work, and approved it for publication.

## Conflict of Interest

The authors declare that the research was conducted in the absence of any commercial or financial relationships that could be construed as a potential conflict of interest.

## Footnotes

1 https://www.wjx.cn/
